# Examining the Spectroscopic and Thermographic Qualities of Er^3+^-doped Oxyfluoride Germanotellurite Glasses

**DOI:** 10.3390/ma15217651

**Published:** 2022-10-31

**Authors:** Witold Ryba-Romanowski, Jarosław Komar, Radosław Lisiecki

**Affiliations:** Institute of Low Temperature and Structure Research, Polish Academy of Sciences, ul. Okólna 2, 50-422 Wrocław, Poland

**Keywords:** luminescence, Er-doped oxyfluoride glass, optical spectroscopy

## Abstract

Novel ternary fluoro-germano-tellurite (GTS) glasses doped with Er^3+^ ions with 0.5 mol% and 1.0 mol% were fabricated by a conventional melt and quenching method and investigated using methods of optical spectroscopy. The room-temperature absorption spectrum was recorded and analyzed to determine radiative transition rates, radiative lifetimes, and branching ratios of Er^3+^ luminescence. Decay curves of Er^3+^ luminesccence were recorded and analyzed. Temperature dependences of emission spectra and absorption spectra in the region from RT (room-temperature) up to 675 K were studied in detail. The contribution of competing radiative and nonradiative processes to the relaxation of luminescent levels of Er^3+^ was assessed. Absolute and relative sensitivity were established utilizing the comprehensive model based on thermally coupled ^2^H_11/2_/^4^S_3/2_ excited states of erbium. The high quantum efficiency of the first erbium-excited state and value of gain coefficient indicate that GTS:Er glass system can be considered as conceivable NIR (near infrared) laser material as well.

## 1. Introduction

Among the lanthanide ions, erbium is one of the most important luminescent centers due to its specific and convenient energy level structure leading to efficient visible and/or NIR emission in the various glass hosts [1,2,3]. Oxyfluoride glasses doped with erbium have been investigated owing to their potential applications as effective NIR optical amplifiers, visible lasers, or temperature sensors [4,5,6,7,8]. For conventional thermometers some serious drawbacks have been found as there is a need to be in touch with the measured target for a period of time to achieve thermal equilibrium [9,10,11]. To overcome this inconvenience, sophisticated approaches have been proposed including temperature dependence of rare-earth ions luminescence [12,13,14]. In the case of the erbium emission, optical temperature sensing can be attributed to temperature-dependent visible emission originating in thermally coupled ^2^H_11/2_ and ^4^S_3/2_ excited states [15,16,17]. The fluorescence intensity ratio (FIR) technique is applicable when alternative erbium luminescence ^4^F_7/2_–^4^I_15/2_ or ^4^F_9/2_–^4^I_15/2_ are utilized [18,19]. As result, numerous potential applications such as temperature monitoring in the industrial factories, electrical power stations, or resource mines may be related to Er-doped luminescent materials [20].

Visible and NIR erbium emissions are significantly affected by the selected glass hosts. The oxyfluoride glasses are recognized as favorable and perspective optical materials since these hosts merge the considerable chemical stability of oxides and luminescence performances of fluorides [21]. For oxyfluoride glasses the impact of fluorine in high quantum yield oxyfluoride glasses and glass-ceramics was investigated by T. Meyneng et. al. [22]. The effect of germanium concentration on visible emission of Er^3+^ in tellurite glasses was studied by O.B. Silva et. al. [23]. It was reported that the quenching effect of OH- group on ^4^I_11/2_ and ^4^I_13/2_ Er^3+^ lifetimes was ineffective for low Er^3+^ content in germanotellurite glasses [24]. The significant thermal stability was documented for erbium-doped germanotellurite glasses for broadband fiber amplifiers [25]. An optical cooling approach based on ^2^H_11/2_ anti-Stokes fluorescence of in Er^3+^-doped tellurite-germanate glasses was proposed [26]. Furthermore, the green up-converted erbium luminescence in germanotellurite glasses was applied to examine the optical sensor qualities [27].

Quite recently, the anti-Stokes erbium luminescence was found to be enhanced with the increase in fluorine concentration in fluorotellurite glasses [28]. The inconvenient thermal effect can take place when up-converted erbium luminescence is initiated by NIR excitation from high-power laser diodes. In relation, we deal with effective down-converted erbium emission excited into intense hypersensitive absorption band at 380 nm. It was believed that a combination of germanium oxide (GeO_2_) and tellurium oxide (TeO_2_) based components with SrF_2_ fluoride modifier may lead to advantageous optical and spectroscopic qualities of Er^3+^ luminescence admixture.

Transition intensities of erbium in germanotellurite glass can be analyzed in the framework of a theory developed independently by Judd and Ofelt. The Judd–Ofelt theory is useful in the interpretation of the absorption spectra of rare earth and it is an excellent research instrument in anticipating relaxation rates of excited levels [29,30,31]. Accordingly, this approach is widely used to assess the luminescent and laser potential of rare-earth-doped glasses as well.

In this work, we report on the spectroscopic properties of a novel fluoro-germano-tellurite (GTS) glasses single-doped with Er^3+^. The radiative and nonradiative transition rates from involved Er^3+^ luminescent levels were analyzed. Optical spectra and the involved erbium-excited states of relaxation were studied as a function of temperature in the range of 300–675 K. Moreover, laser operation around 1.55 μm was assessed examining potential resonant pump process and gain coefficient as well.

## 2. Materials and Methods

Ternary fluoro-germano-tellurite (GTS) glasses examined in the present work were fabricated by a conventional melt and quenching method. The starting materials are comprised of a few reagents as follows: GeO_2_, TeO_2_, SrF_2_, and high purity 99.99 Er_2_O_3_. The final glass compositions in mol% can be expressed as 49.5GeO_2_–35TeO_2_–15SrF_2_–0.5Er_2_O_3_ and 49GeO_2_–35TeO_2_–15SrF_2_–1Er_2_O_3_. The reagents were thoroughly mixed and grinded in an atmosphere of argon in a MBRAUN glovebox (M. BRAUN INCORPORATED, Stratham, NH 03885, USA)(water content ≤1 ppm) and the prepared batches (8 g) were melted at 920 °C using an electric furnace in a corundum crucible in ambient atmosphere for 1 h. The obtained GTS glasses were slowly annealed and next were cut and accurately polished for the specified experiments.

Survey absorption spectra of the Er^3+^-doped germanotellurite glasses were recorded in the 300–1800 nm spectral region utilizing an Agilent Cary 5000 UV-vis-NIR spectrophotometer (Agilent, 5301 Stevens Creek Blvd, Santa Clara, CA 95051, USA). For the temperature-dependent absorption spectra, the glass samples were mounted into a chamber furnace which was next assembled at the spectrophotometer. The applied resolution was 0.5 nm in NIR and 0.2 nm in UV-Vis. Part of the emission spectra were measured using a 380 nm excitation and recorded using an FLS980 steady-state fluorescence spectrometer (Edinburgh Instruments Ltd. 2 Bain Square, Kirkton Campus, EH54 7DQ, UK) equipped with 928 Hamamatsu photomultiplier (Hamamatsu, 430-0852 2-25-7 Ryoke, Naka-ku, Japan) and InGaAs detector (Hamamatsu, 430-0852 2-25-7 Ryoke, Naka-ku, Japan). For the temperature-dependent emission measurements, the sample was placed in a chamber furnace and the UV-vis and NIR luminescence was recorded with a DongWoo DM711 monochromator (DongWoo Optron Co. Ltd., Kyungg-do, Korea). The copper-constantan thermocouple was used to measure the GTS:Er glass temperature which is thoroughly controlled by a proportional-integral-derivative (PID) Omron E5CK controller (OMRON Corporation, Kyoto, Japan). The decay curves of erbium luminescent levels were measured utilizing a Tektronix 350 MHz MSO-44 Mixed Domain Oscilloscope (Tektronix Inc, Beaverton, OR 97077, USA) and these transients were selectively excited by a Continuum Surelite 1 optical parametric oscillator (OPO) (Amplitude Laser Group, San Jose, CA, USA) pumped with a third harmonic of a 4-ns pulse Nd:YAG laser.

## 3. Results and Discussion

### 3.1. XRD Examination

**Figure 1:**
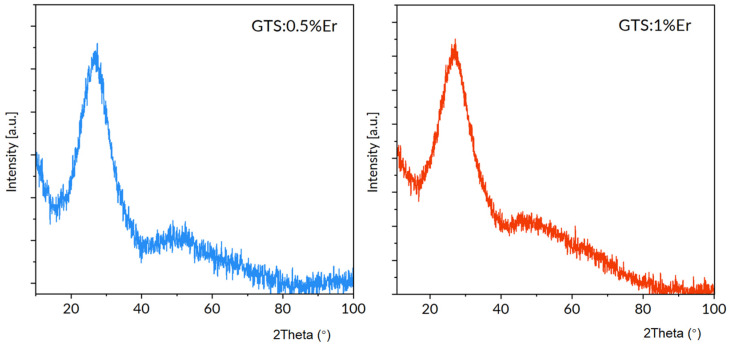
XRD patterns of GTS: 0.5% Er and GTS: 1% Er glasses.

Figure 1 presents the XRD patterns of GTS: 0.5% Er; and GTS: 1% Er germanatetellurite glasses. The multicomponent amorphous materials are examined, hence various phases can appear during fabrication process. To verify glass phase purity, XRD diffractograms were recorded as depicted in Figure 1. It is perceived that quite broad amorphous bands occur, and no sharp and narrow lines exist in the studied XRD patterns. Therefore, it can be concluded that no crystallization takes place after the melt solidification process.

### 3.2. Absorption Spectra and Judd–Ofelt Calculations

**Figure 2:**
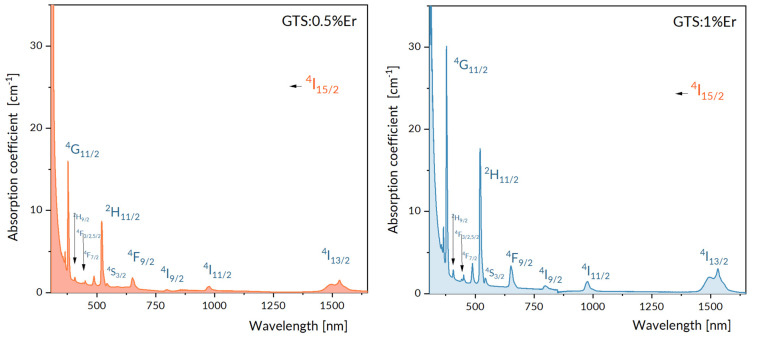
Survey absorption spectra recorded at room temperature for GTS: 0.5% Er and GTS: 1% Er samples.

Figure 2 shows a survey absorption spectrum recorded at room temperature for GTS: 0.5% Er and GTS: 1% Er samples. It can be seen in Figure 2 that intensities of absorption transitions located at wavelength above about 750 nm are small, implying possible calculation incertitude; therefore, we analyzed the following absorption bands acquired for GTS: 1% Er sample. Terminal multiplets involved in transitions subjected to the Judd–Ofelt treatment are assigned in this figure. A set of nine absorption bands related to transitions from the ^4^I_15/2_ ground state to low energy excited levels of Er^3+^ that contribute to the absorption spectrum between 1700 nm and 400 nm was subjected to routine Judd–Ofelt (J–O) treatment. Adopting this approach, the values of experimental oscillator strengths P_exp._ were evaluated by numerical integration of corresponding absorption bands for GTS: 1% Er assigned in Figure 2.

Erbium absorption bands in GTS: 1% Er were applied to calculate the experimental oscillator strengths applying the following relation [32]:

(1) $$P_{exp.}=\frac{mc}{\pi e^{2}N}\int \alpha \left(\nu \right)d\nu$$

where the *α(ν)dν* means an integrated area at a wavenumber *ν*, *m* [g] is electron mass, *e* [statC] is the electron charge, and *N* [ions/cm^3^] is the Er^3+^ concentration.

In the Judd–Ofelt theory, the oscillator strength of an electric-dipole transition between initial state *J* and terminal one *J*’ is defined as [32]:

(2) $$P_{theor.}=\frac{8\pi^{2}mc}{3h\lambda \left(2J+1\right)}\frac{{\left(n^{2}+2\right)}^{2}}{9n}\underset{t}{\sum }\Omega_{t}{\left|4f^{n}\left\langle [S,L\right]J||L+2S||\left[S^{\prime },L^{\prime }\right]J^{\prime }\rangle \right|}^{2}$$

where *λ* is the mean wavelength of the transition and *n* is the refractive index of the host.

For each transition the theoretical oscillator strength *P_theor._* is constructed as a sum of products of matrix elements of unit tensor operator <U^*t*^> and three phenomenological Ω_*t*_ parameters. Next, the values of Ω_*t*_ are obtained by a least square fit between *P_exp._* and *P_theor_.* Results of the fitting procedure are presented in Table 1 and obtained Ω parameters are as follows:

Values of radiative transition rates W_r_, luminescence branching ratios β, and radiative lifetimes τ_rad._ evaluated for low energy excited states of Er^3+^ ions in GTS glass using Ω_*t*_ parameters are gathered in Table 2.

### 3.3. Relaxation Dynamic of Excited States

It is worth comparing the value W_r_ = 2998 s^−1^ calculated above for the ^4^S_3/2_ multiplet to corresponding data reported for erbium-doped glasses of concern. For instance, W_r_(^4^S_3/2_) = 3394 s^−1^ has been reported for multicomponent tellurite glass [12], W_r_(^4^S_3/2_) = 3058 s^−1^ has been reported for multicomponent germanotellurite glass [23], and W_r_(^4^S_3/2_) = 2570 s^−1^ has been reported for multicomponent fluorotellurite glass [28]. This comparison implies that W_r_(^4^S_3/2_) depends on the host glass in rather moderate degree. However, the markedly higher value W_r_(^4^S_3/2_) = 4464 s^−1^ has been reported for heavy metal oxyfluoride PbF_2_-AlF_3_-TeO_2_ glass [33].

The energy difference between the ^4^S_3/2_ and ^2^H_11/2_ levels is sufficiently small to assure their thermalisation, i.e., their population of is consistent with the Boltzmann statistics and thereby the two levels decay with a common radiative rate W_therm_ provided by the formula [12,34]:

(3) $$W_{\mathrm{therm}.}=\frac{12\cdot W_{2_{H_{11/2}}}\;\exp\left(\frac{-\Delta E}{k_{B}T}\right)+(4\cdot W_{4_{S_{3/2}}})}{12\exp\left(\frac{-\Delta E}{k_{B}T}\right)+4}$$

where *W* denotes radiative rates values of levels involved, Δ*E* is their energy difference, and *T* denotes the temperature and *k_B_* denotes Boltzmann constant. In principle, a decay rate *W* of an excited state of rare-earth ions is a sum of competing radiative rates *Wr.*, multiphonon relaxation rates *W_mph_*., and decay rates of nonradiative energy transfer *W_q_*_._ between interacting rare-earth ions. An ability of an excited ion to provide luminescent transition is assessed by a quantum efficiency *η* defined as [34]:

*η* = *W_r_*/(*W_r_* + *W_mph_* + *W_q_*) (4)

Introducing a concept of lifetime *τ* defined as *τ* = 1/*W_ij_* the quantum efficiency for an excited state can be expressed also as *η = τ_lum._/τ_rad_* where *τ_lum._* is luminescence lifetime determined commonly based on analysis experimental curves of luminescence decay following a short pulse excitation. Table 3 gathers luminescence lifetime values determined from decay curves of luminescence recorded at room temperature for samples GTS: 0.5% Er and GTS: 1% Er.

It can be seen that Er concentration in the samples affects weakly lifetime values except for that of the ^2^H_11/2_, ^4^S_3/2_ pair of levels.

**Figure 3:**
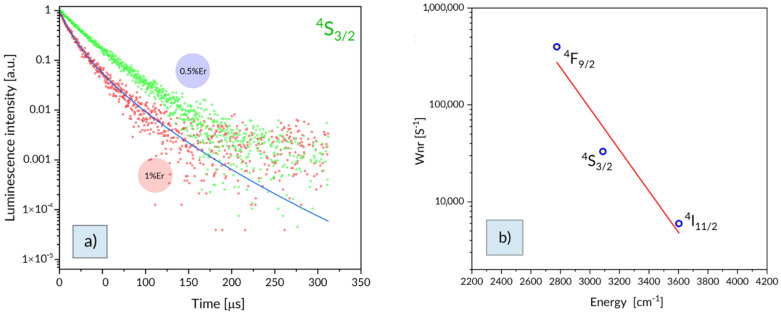
Decay curves of ^4^S_3/2_ luminescence (**a**) and estimated nonradiative relaxation rates versus related energy gap (**b**). Solid line indicates the theoretical energy gap dependence provided by Equation (8).

Decay curves of the ^2^H_11/2_, ^4^S_3/2_ luminescence, compared in Figure 3, indicate that the decay curve for higher Er concentration show significant deviation from single exponential time dependence. Therefore, the corresponding lifetime value in Table 3 is a mean lifetime value *τ_avg._* calculated using a relation [35]:

(5) $$\tau_{avg.}=\frac{\int tI\left(t\right)dt}{\int I\left(t\right)dt}$$

Observed effect of Er concentration on luminescence decay curve points at the onset of the contribution of a decay related to ion–ion interaction. In fact, there is a good agreement between the experimental decay curve for GTS: 1% Er sample and a solid line in Figure 3, representing the time dependence predicted by the Inokuti–Hirayama relation [36]:

(6) $$\Phi \left(t\right)=Aexp\left[-\left(\frac{t}{\tau_{0}}\right)-\alpha {\left(\frac{t}{\tau_{0}}\right)}^{3/S}\right]$$

where *Φ(t)* denotes emission intensity after pulse excitation, *A* is constant, *S* = 6 for dipole–dipole interactions between erbium ions, *τ_0_* is the intrinsic decay probability of the donor involved in energy transfer process in the absence of acceptor, and *α* parameter is defined as:

(7) $$\alpha =\left(\frac{4}{3\pi }\right)\Gamma \left(1-\frac{3}{S}\right)N_{a}R_{0}^{3}$$

where *N_a_* is acceptor concentration, Γ = 1.77 (for *S* = 6) is the Euler’s function, and *R_0_* is the critical ion–ion distance at which the probability of donor–acceptor energy transfer process is consistent with the intrinsic decay rate *τ*_0_. Our Er-doped GTS glass system ET phenomena corresponds to cross-relaxation processes among erbium luminescent activators. 

Presented on Figure 3a the non-exponential decay curve of ^4^S_3/2_ luminescence measured for GTS: 1% Er glass was adequately fitted and the appropriate results were achieved for S = 6 and α = 1.92. The critical transfer distance was determined to be R_0_ = 10.7 Å. The donor–acceptor interaction parameter provided by the relation C_da_ = R_0_^6^τ_0_^−1^ was found to be 4.56 × 10^−51^ m^6^s^−1^.

Radiative transition rates and radiative lifetimes gathered in Table 2 and luminescence lifetimes for GTS: 0.5% Er sample presented in Table 3 made it possible to evaluate quantum efficiencies and contribution of multiphonon relaxation rates to luminescence from the ^2^H_11/2_, ^4^S_3/2_, ^4^F_9/2_, and ^4^I_11/2_ levels. In Figure 3a the rates of multiphonon relaxation W_nr._ obtained subtracting the W_r_ values from the inverse of luminescence lifetime of respective levels are indicated by points. Solid line represents a so-called energy gap dependence according to relation [37]:

W_nr_ = Cexp(-αΔE) (8)

where C = 2.23 × 10^11^ s^−1^ and α = 4.89 × 10^−3^ cm^−1^are parameters characteristic of the host on the study.

In an early study Reisfeld and Eckstein [38] investigated quantum efficiency of visible luminescence of Er^3+^ ions in tellurite and germanate glasses. Multiphonon relaxation rate of the ^4^S_3/2_–^4^F_9/2_ transition was then found to be 1.16 × 10^4^ s^−1^ for tellurite glass and 1.60 × 10^5^ s^−1^ for germanate glass. The rate of the ^4^F_9/2_–^4^I_9/2_ transition was then found to be 2.85 × 10^5^ s^−1^ for germanate glass and 2.33 × 10^5^ s^−1^ for tellurite glass. The higher rates for germanate glasses have been attributed to stronger ion–lattice interaction. Multiphonon relaxation rate amounting to 3.3 × 10^4^ s^−1^ and to 3.97 × 10^5^ s^−1^ was calculated for the ^4^S_3/2_ and the ^4^F_9/2_, respectively, for our glass samples are consistent with those reported in the past.

### 3.4. Emission and Thermographic Qualities

**Figure 4:**
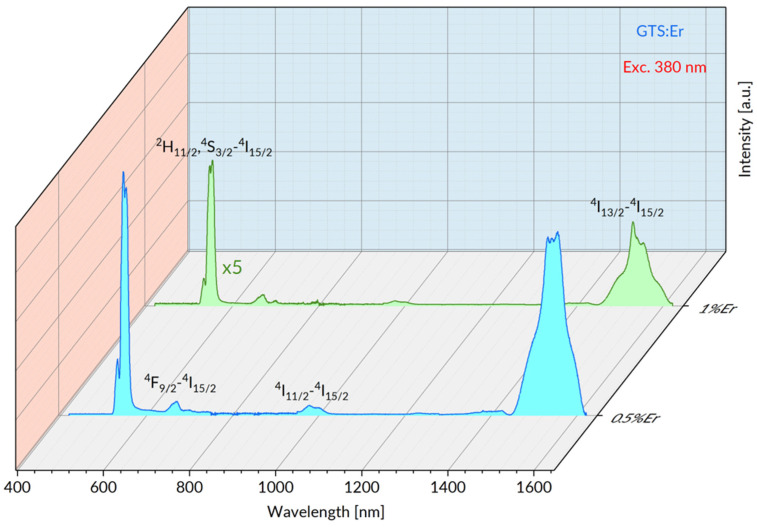
Vis-NIR emission spectra of GTS: 0.5% Er and GTS: 1% Er glasses excited at 380 nm.

Figure 4 shows survey emission spectra of GTS: 0.5% Er and GTS: 1% Er glasses measured within 400–1650 nm upon excitation at 380 nm.

The luminescence originating in ^4^S_3/2_ and ^4^F_9/2_ excited states contributes to the visible spectral range and the ^4^I_11/2_–^4^I_15/2_ and ^4^I_13/2_–^4^I_15/2_ transitions appear in near infrared. It follows from these spectra that integrated intensity of GTS: 1% Er luminescence is already significantly lowered in relation to GTS: 0.5% Er sample. Results presented above deserve some comments. The concentrations of the optically active ions 0.5% Er and 1% Er considered in this study represent a trade-off between required pumping efficiency and the highest possible intensity of luminescence free from adverse phenomena consisting of (i) concentration quenching by non-radiative ion–ion interaction and (ii) self-absorption of emitted photons. In principle, absorption efficiency increases with increasing Er concentration, but the growing contribution of the adverse concentration quenching reduces strongly the ^2^H_11/2_, ^4^S_3/2_ emission intensity. It is shown in the following figure that the phenomenon of self-absorption affects adversely the ^4^I_13/2_–^4^I_15/2_ luminescence band intensity.

**Figure 5:**
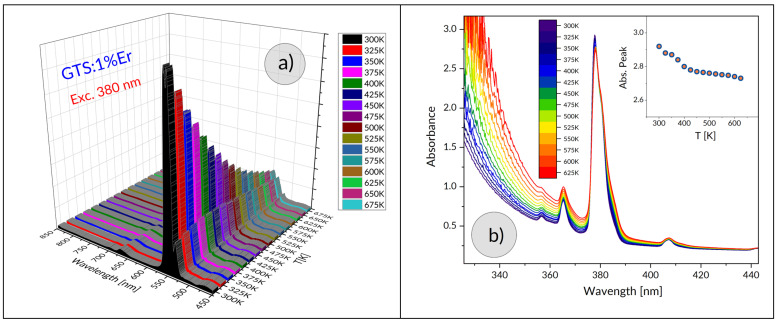
The ^2^H_11/2_, ^4^S_3/2_ emission band (**a**) and the ^4^I_15/2_–^4^G_11/2_ absorption band (**b**) for GTS: 1% Er glass measured as function of temperature. Inset shows a plot of peak intensity of the the ^2^H_11/2_, ^4^S_3/2_ absorption versus temperature for the band located at about 380 nm serving for optical pumping.

The thermally induced change in the shape of the luminescence band related to transitions from the ^2^H_11/2_, ^4^S_3/2_ multiplets in thermal equilibrium is presented in Figure 5a.

**Figure 6:**
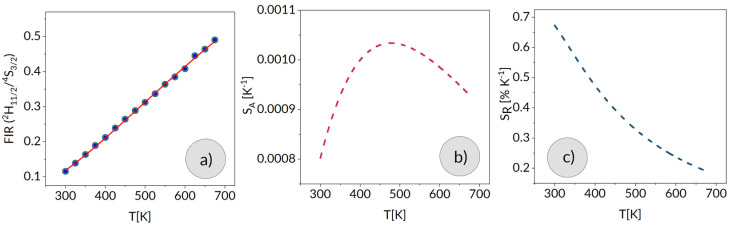
Fluorescence intensity ratio (^2^H_11/2_/^4^S_3/2_) (**a**) as well as estimated absolute (**b**) and relative (**c**) temperature sensitivity.

The emission intensities are proportional to the population of two thermally coupled levels involved and their intensity ratio commonly denoted as fluorescence intensity ratio (FIR) is defined by following equation [18]:

(9) $$FIR=\frac{I_{{}^{2}H_{11/2}}}{I_{{}^{4}S_{3/2}}}=B\exp\left(-\frac{\Delta E}{kT}\right)$$

where *B* is the temperature-independent constant, Δ*E* is the energy gap between the two thermally coupled levels, and *k* is the Boltzmann constant. Figure 6 shows a plot of FIR versus temperature for the luminescence in question. Experimental data are indicated by points and a solid line represents a fit of Equation (9) with Δ*E* = 659 cm^−1^.

The absolute FIR change *S_A_* with temperature variation expressed as [18]:

(10) $$S_{A}=\frac{dFIR}{dT}=FIR\frac{\Delta E}{kT^{2}}$$

and its relative value *S_R_*

(11) $$S_{R}=\frac{1}{FIR}\frac{dFIR}{dT}\cdot 100\%=\frac{\Delta E}{kT^{2}}\cdot 100\%$$

are plotted versus temperature in Figure 6b,c, respectively. The *S_A_* and *S_R_* parameters determine thermosensitive properties of a luminescent material and are commonly used to assess its suitability for optical thermometry. For a comparison purposes the *S_A_* and *S_R_* parameters for Er in several glasses based on tellurium or germanium oxides are listed in Table 4.

**Figure 7:**
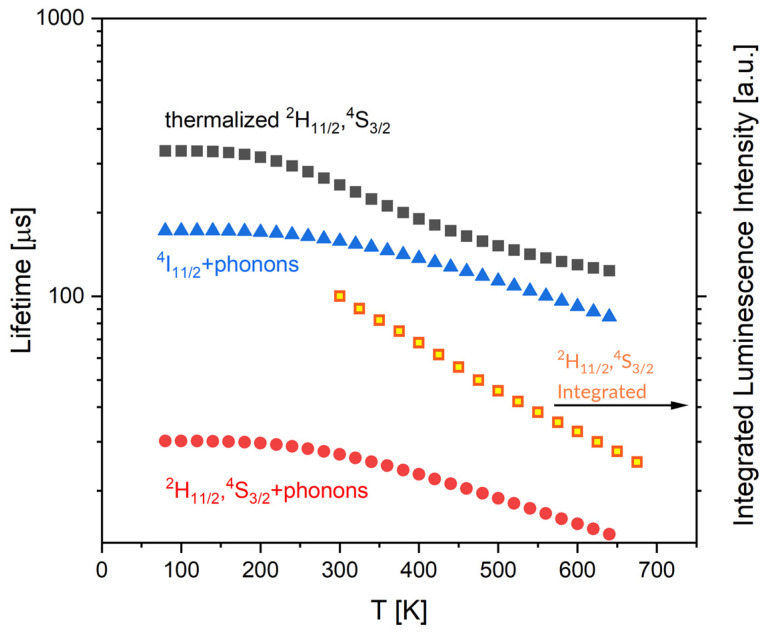
Estimated impact of temperature on radiative transition rate of the ^2^H_11/2_, ^4^S_3/2_ group of levels (■), on luminescence lifetime of the ^4^I_11/2_ level (Δ), on luminescence lifetime of the ^2^H_11/2_, ^4^S_3/2_ group of levels (•) and on integrated luminescence intensity originated in the ^2^H_11/2_, ^4^S_3/2_ group of levels (□).

It can be seen in Figure 5a that the luminescence intensity decreases with increasing temperature. The integrated luminescence intensity originated in the ^2^H_11/2_, ^4^S_3/2_ group of levels (▪) (hollow squares) is plotted versus temperature in Figure 7. It shows that the luminescence intensity normalized to 100 at 300 K decrease to about 20 at 675 K. The origin of this adverse thermal effect is not obvious. The data shown in Figure 5b indicate that the peak value of absorbance diminishes from about 2.9 to 2.7 when the temperature changes from 300 K to 625 K. Accordingly, the observed decrease in the luminescence intensity cannot be attributed to the decrease in the absorption efficiency. It should be noticed here that effect of temperature on the luminescence lifetime *τ* is consistent with a relation [34]:

(12) $$\tau ={\left[\frac{12W_{r}\left({}^{2}H_{11/2}\right)exp\left(-\frac{\Delta E}{k_{B}T}\right)+4W_{r}\left({}^{4}S_{3/2}\right)}{12exp\left(-\frac{\Delta E}{k_{B}T}\right)+4}+W{\left(\frac{exp\frac{\hbar \omega }{k_{B}T}}{exp\frac{\hbar \omega }{k_{B}T}-1}\right)}^{p}\right]}^{-1}$$

where *W_r_* denotes radiative lifetime values of levels involved, *k* is the Boltzmann constant, and Δ*E* is the energy gap between the involved levels.

The first member of the sum on the right hand expresses the radiative transition rate for ^2^H_11/2_, ^4^S_3/2_ group of levels (Equation (1)). It increases with increasing temperature. The second member expresses the multiphonon relaxation rate. It increases with the growing temperature, too. Thus, the quantum efficiency of the ^2^H_11/2_, ^4^S_3/2_ group of levels for each temperature point is governed by the multiphonon relaxation rate diminished by radiative transition rate. The plot of the lifetime *τ* versus temperature in agreement with Equation (12) above is shown in Figure 7 (full red circles).

### 3.5. NIR Spectra and Gain Cross-sections

**Figure 8:**
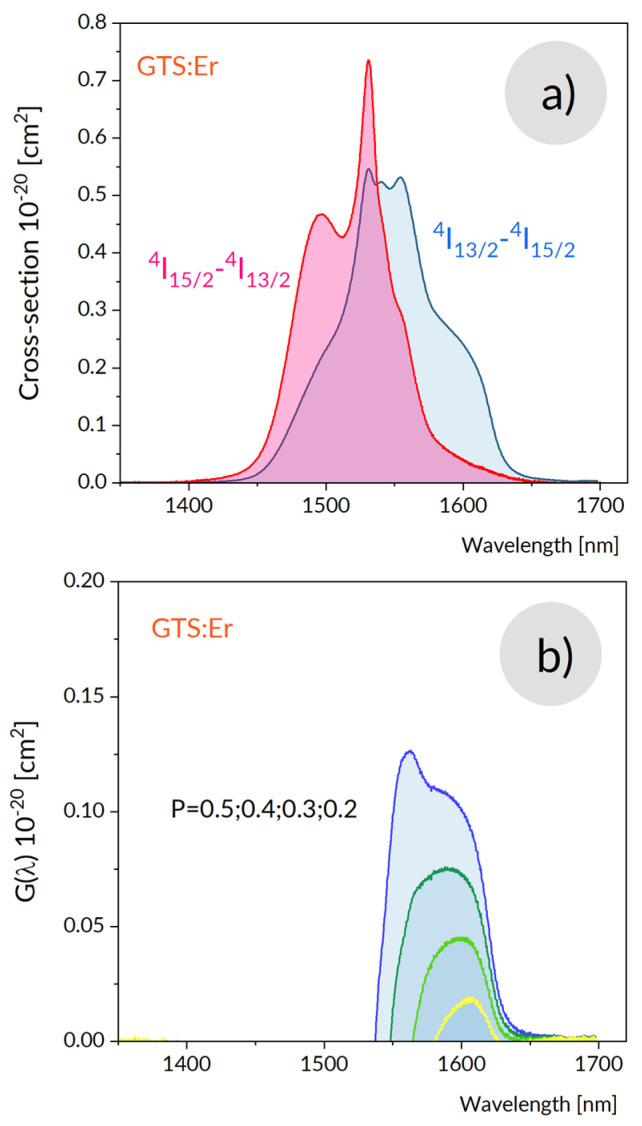
Absorption ^4^I_15/2_-^4^I_13/2_ and emission ^4^I_13/2_–^4^I_15/2_ cross-sections (**a**) and gain cross section determined for several population inversion parameters (**b**).

Another point of interest that appears in studies of erbium-doped tellurite or germanate glasses was related to a potential for laser operation and/or amplification ability in NIR region around 1500 nm. Figure 8a shows the absorption band corresponding to the ^4^I_15/2_–^4^I_13/2_ transition of Er^3+^ in GTS glass recorded at room temperature. The band intensity was calibrated in units of absorption cross-section σ_abs._ defined as the ratio of the absorption coefficient to the density of Er^3+^ ions. The band stretches from about 1395 nm to 1600 nm and shows a main maximum at 1531 nm and a second, less pronounced maximum at 1495 nm.

The band related to the ^4^I_13/2_–^4^I_15/2_ luminescence transition was calculated employing the Fuchtabuer–Ladenburg relation [43]:

(13) $$\sigma_{em.}\left(\lambda \right)=\frac{\beta \lambda^{5}I\left(\lambda \right)}{8\pi n^{2}c\tau_{r}\int \lambda I\left(\lambda \right)d\lambda }$$

where I(λ)/∫λI(λ)dλ is the normalised lineshape function of the experimental emission spectrum *I(λ)*, luminescence branching ratio *β* = 1 for ^4^I_13/2_–^4^I_15/2_ Er^3+^ transition, *τ_r_ (s)* is the ^4^I_13/2_ excited state radiative lifetime, *n* is the refractive index, and *c (m/s)* is the velocity of light.

The calculated band is compared to the ^4^I_15/2_–^4^I_13/2_ absorption band in Figure 8a. It can be seen that the absorption and luminescence bands overlap each other. Nevertheless, the emission intensity exceeds the absorption intensity in the long wavelength part of the spectra pointing at the ability to the light amplification. The predicted gain coefficient *G(λ)* accounting for re-absorption losses is provided by the relation:

(14) $$G\left(\lambda \right)=N\left[P\sigma_{em.}\left(\lambda \right)-\left(1-P\right)\sigma_{abs.}\left(\lambda \right)\right]\;$$

where *P* is the population inversion parameter defined as the ratio of density of Er ions in excited state to a total density *N* of Er ions in the glass. *G(λ)* values calculated using the relation (14) above for several values of *P* is plotted versus wavelength in Figure 8b. It follows from this plot that the laser action in a free operation regime occurs at 1682 nm (as indicated by a positive *G(λ)* value for the lowest value of (*P*)). It can be seen also that with a dispersive element inserted to a laser cavity a tunable laser operation within a region dependent on the *P* parameter would be possible. The main shortcomings are thermal effects that adversely affect a laser potential of a glassy active media characterized by relatively small thermal conductivity. In fact, for commonly applied optical pumping into the ^4^I_11/2_ absorption band there is a huge quantum defect, i.e., energy difference between a pump photon and a photon emitted in the laser transition that heat laser active material prevents, thereby a laser performance at required power levels. To overcome this shortcoming the optical pumping with diode lasers emitting at 1500 nm would match perfectly a second absorption maximum of GTS: Er glass providing efficient resonant optical pumping with a marginal quantum defect [44,45].

## 4. Conclusions

Samples of ternary fluoro-germano-tellurite glass containing Er^3+^ ions with concentration of 0.5 mol% and 1.0 mol% were investigated. Comparison of the calculated value of radiative transition rate W_r_ = 2998 s^−1^ for the ^4^S_3/2_ multiplet to corresponding data reported thus far indicate that it depends weakly on the change in composition for various tellurite and germanate host glasses. Rates of multiphonon relaxation of the ^4^S_3/2_ and ^4^F_9/2_ are consistent with those reported in the past for Er^3+^-doped tellurite and germanate glasses. The onset of the self-quenching of the ^2^H_11/2_, ^4^S_3/2_ luminescence occurs at Er^3+^ concentration below 1 mol%. The integrated luminescence intensity originated in the ^2^H_11/2_, ^4^S_3/2_ group of levels decreases roughly by a factor of five when the temperature increases from 300 K to 625 K. Interpretation of this finding based on assessment of temperature dependence of radiative transition rates and of multiphonon relaxation provides a reasonable qualitative justification. However, the contribution of temperature-dependent quenching by a nonradiative energy transfer mechanism is likely to occur. A high rate of multiphonon relaxation for the ^4^I_11/2_ pump level is advantageous for laser operation around 1680 nm since it reduces parasitic radiative transition to the ground state. However, a huge quantum defect related to this excitation way is a serious shortcoming. In fact, for commonly applied optical pumping into the ^4^I_11/2_ absorption band around 980 nm there is a huge quantum defect, i.e., energy difference between pump photon and a photon emitted in the laser transition that heat laser active material preventing laser performance at practically required power levels. To overcome this shortcoming the diode lasers emitting at about 1500 nm can be used for optical pumping. The peculiarity of the ^4^I_15/2_–^4^I_13-2_ absorption transition band showing a well-defined second maximum at 1495 nm in GTS: Er glass offers a way for resonant optical pumping into the upper laser level with a marginal quantum defect.

## Figures and Tables

**Table 1:** Experimental and theoretical oscillator strengths of Er^3+^ transitions in GTS glass.

^4^I_15/2_ → ^2S+1^L_J_	Energy [cm^−1^]	Oscillator Strength P_p_ [×10^−6^]		
		P_exp._	P_theor._	∆P
^4^I_15/2_	6592	1.97 (ED), 0.72 (MD)	1.98	0.01
^4^I_11/2_	10,241	1.03	1.01	0.02
^4^I_9/2_	12,496	0.48	0.55	0.07
^4^F_9/2_	15,293	3.32	3.24	0.08
^4^S_3/2_	18,379	0.63	0.71	0.08
^2^H_11/2_	19,164	16.80	16.81	0.01
^4^F_7/2_	20,474	2.66	2.98	0.32
^4^F_3/2_,_5/2_	22,169	1.07	1.36	0.29
^4^H_9/2_	24,583	0.96	1.08	0.12
Ω_2_ = 9.35 × 10^−20^ cm^2^,Ω_4_ = 2.46 × 10^−20^ cm^2^,Ω_6_ = 1.33 × 10^−20^ cm^2^ RMS = 3.21 × 10^−7^				

**Table 2:** Radiative transition rates W_r_, luminescence branching ratios β, and radiative lifetimes τ_rad._ for Er^3+^ ions in GTS glass.

SLJ	*S’L’J’*	Wr [s^−1^]	β	τ_rad._ [µs]
^4^I_13/2_	^4^I_15/2_	207.14	1.00	4828
^4^I_11/2_	^4^I_15/2_	295.43	0.89	3026
	^4^I_13/2_	35.03	0.11	
^4^I_9/2_	^4^I_15/2_	290.95	0.77	2631
	^4^I_13/2_	87.41	0.23	
	^4^I_11/2_	1.66	0.00	
^4^F_9/2_	^4^I_15/2_	2541.96	0.90	355
	^4^I_13/2_	133.19	0.05	
	^4^I_11/2_	130.79	0.05	
	^4^I_9/2_	10.29	0.00	
^4^S_3/2_	^4^I_15/2_	2002.67	0.67	334
	^4^I_13/2_	822.40	0.27	
	^4^I_11/2_	63.15	0.02	
	^4^I_9/2_	108.27	0.04	
	^4^F_9/2_	1.16	0.00	
^2^H_11/2_	^4^I_15/2_	17,255.60	0.96	55
	^4^I_13/2_	269.57	0.01	
	^4^I_11/2_	157.15	0.01	
	^4^I_9/2_	238.10	0.01	
	^4^F_9/2_	64.42	0.01	
	^4^S_3/2_	0.07	0.00	

**Table 3:** Experimental lifetimes of luminescent levels of Er^3+^ ions in germanotellurite glasses GTS: Er.

Sample	Lifetime [µs]			
	^4^S_3/2_	^4^F_9/2_	^4^I_11/2_	^4^I_13/2_
GTS: 0.5% Er	27.1	2.5	159	3935
GTS: 1% Er	14.8	2.2	142	3395

**Table 4:** Comparison of absolute and relative sensitivities estimated for Er-doped glass system.

Glass	Temperature [K]	S_A_ [K^−1^]	S_R_ [%K^−1^]	Ref.
TeO_2_–ZnO–ZnF_2_–La_2_O_3_–Yb_2_O_3_–Er_2_O_3_	300 K	0.0015	1.21	[28]
GeO_2_-PbO-Ga_2_O_3_-Er_2_O_3_	300 K	0.0035	0.98	[39]
TeO_2_-GeO_2_-PbO-PbF_2_-BaO-LaF_3_-Er_2_O_3_	300 K	0.0022	0.70	[40]
TeO_2_-PbCl_2_-WO_3-_Er_2_O_3_	300 K	0.0030	1.20	[41]
TeO_2_-PbF_2_-AlF_3_-Er_2_O_3_	300 K	0.0033	1.19	[42]
GeO_2_-TeO_2_-SrF_2_-Er_2_O_3_	300 K	0.0008	0.67	This work

## Data Availability

The data presented in this study are available on request from the corresponding author.
